# Safety and efficacy of cervical 10 kHz spinal cord stimulation in chronic refractory primary headaches: a retrospective case series

**DOI:** 10.1186/s10194-016-0657-2

**Published:** 2016-07-08

**Authors:** Giorgio Lambru, Michele Trimboli, Stefano Palmisani, Thomas Smith, Adnan Al-Kaisy

**Affiliations:** The Headache Centre, Pain Management and Neuromodulation Department, Guy’s and St Thomas’ NHS Foundation Trust, London, UK

**Keywords:** Chronic migraine, Cluster headache, SUNA syndrome, Neurostimulation, Spinal cord stimulation, Refractory headaches

## Abstract

**Background:**

Paresthesia-free cervical 10 kHz spinal cord stimulation (HF10 SCS) may constitute a novel treatment modality for headache disorders, when pharmacological approaches fail. We report the results of a retrospective analysis assessing the long-term safety, tolerability and efficacy of HF10 SCS in a group of patients with chronic refractory primary headache disorders.

**Findings:**

Four patients with chronic migraine (CM), two with chronic SUNA (Short-lasting Unilateral Neuralgiform headache attacks with Autonomic symptoms) and one with chronic cluster headache (CCH) refractory to medical treatments, were implanted with cervical HF10 SCS. Pre- and post-implantation data were collected from the medical notes and from headache charts. At an average follow-up of 28 months (range: 12–42 months) we observed an improvement of at least 50 % in headache frequency and/or intensity in all CM patients. One SUNA patient became pain free and the other reported at least 50 % improvement in attacks frequency an duration. The CCH patient reported a significant reduction in CH attacks duration. Two patients underwent a surgical revision due to lead migration.

**Conclusions:**

Paresthesia-free high cervical HF10 SCS appears to be a long-term safe and likely effective therapeutic approach for patients with chronic refractory primary headache disorders. These results warrant further prospective studies in larger series of patients.

## Background

Chronic daily headache (CDH) is an umbrella term that refers to headache disorders that occur on 15 or more days per month for more than 3 months. Primary CDH is a major worldwide health problem that affects 3–5 % of the population [1]. Chronic migraine (CM) is the most common and disabling primary CDH [2, 3]. Less frequent, but possibly more disabling primary CDH conditions include the chronic forms of trigeminal autonomic cephalalgias (TACs). This group encompasses cluster headache (CH), short-lasting unilateral neuralgiform headache attacks with conjunctival injection and tearing (SUNCT), short-lasting unilateral neuralgiform headache attacks with cranial autonomic features (SUNA), paroxysmal hemicrania (PH) and hemicrania continua (HC) [4].

Despite advances in the management of headache disorders a significant minority of patients fails to respond or tolerate conventional medical approaches, falling into the category of medically “refractory” headaches. The definition of refractory headaches is an ongoing matter of debate and numerous attempts to draw a consensus have been proposed [5, 6]. For this group of patients, neurostimulation therapies targeting peripheral or central nervous system structures have been emerging as potential treatments [7]. Occipital nerve stimulation (ONS) has been used as first line neurostimulation treatment for the management of various primary headache disorders, including CM, chronic CH (CCH), HC, SUNCT and SUNA [8, 9], based on the encouraging experience of open-label studies. However three large randomized controlled trials (RCTs) testing the efficacy of ONS for CM prevention, displayed modest efficacy, although the studies were criticized for poor methodological designs [10–12].

High frequency (10Khz) spinal cord stimulation (HF10 SCS) is a paresthesia-free neurostimulation therapy, which has been shown to be effective in some chronic pain conditions [13–15]. In view of the mixed outcome of ONS, we began offering high cervical HF10 SCS as part of our neurostimulation approaches to patients with medically refractory chronic primary headache syndromes and participated in a prospective exploratory study testing cervical HF10 SCS in 17 refractory CM. The study showed promising results in terms of tolerability and effectiveness of the therapy [16]. We report here the long-term follow-up of the patients treated in our headache centre with cervical HF10 SCS.

## Methods

### Participants

Seven patients with primary CDH were considered suitable candidates for neurostimulation and were offered a trial of HF10 SCS therapy. All patients were diagnosed according to the International Headache Society (IHS) classification criteria [17] by two, experienced, headache neurologists (SC and GL). Four had CM, two patients had chronic SUNA and one patient had CCH. Patients were considered refractory to conventional pharmacological treatments, according to the proposed definition at the time of screening [5]. Botulinum toxin type A (Botox®) was also tried according to the PREEMPT paradigm [18] in three out of four CM patients, before considering them refractory [6]. All patients had highly disabling headaches according to the Headache Impact Test (HIT-6). As part of our neurostimulation pathway, patients clinically suitable for invasive neurostimulation attend a cognitive behavioural therapy based implant preparation programme [19]. Six out of seven patients completed the programme before undergoing the stimulation trial. This study was an audit of outcome and, as such under UK guidelines, did not require ethics committee approval.

### Implant procedure

**Fig. 1:**
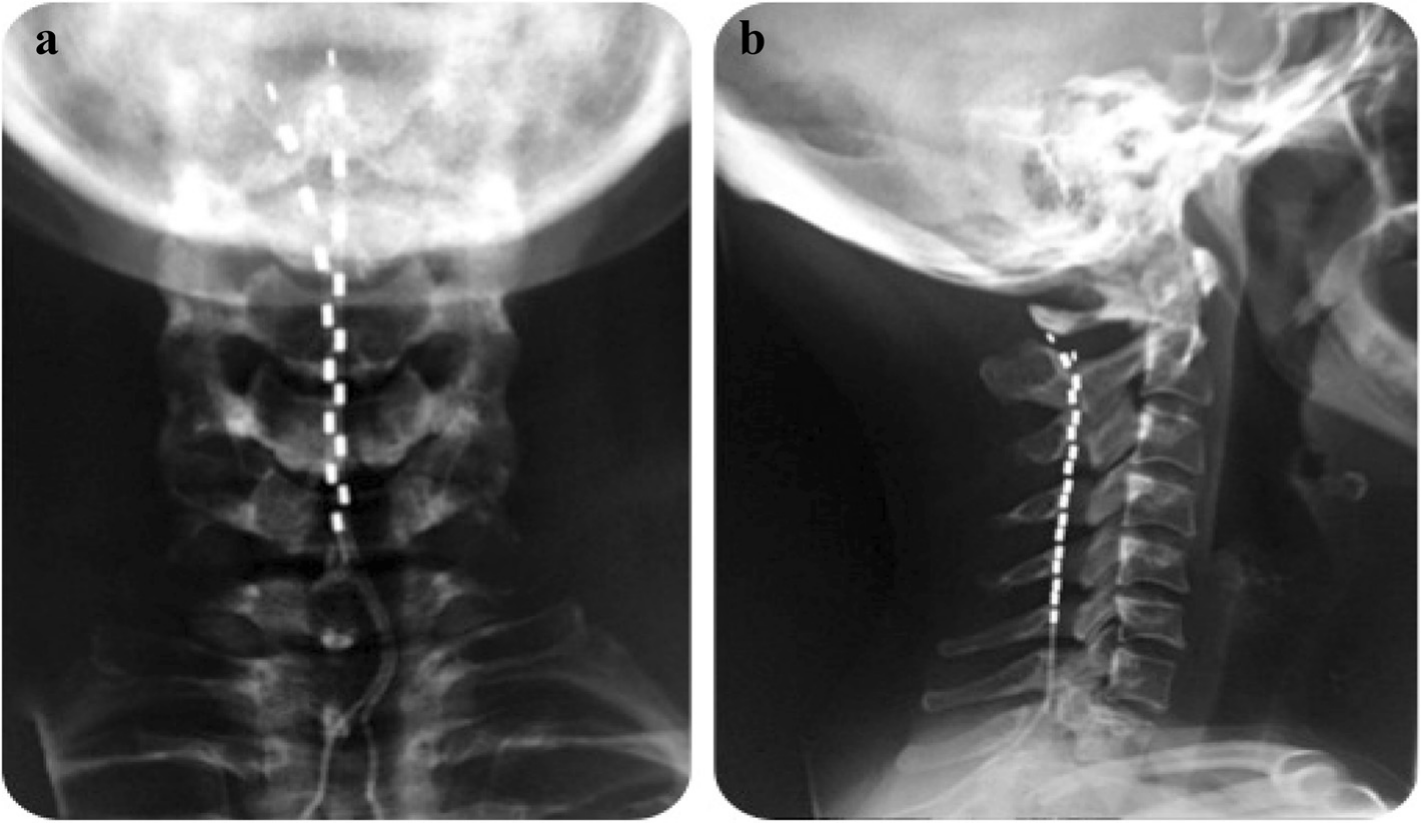
Antero-posterior (**a**) and lateral (**b**) radiographic view of the cervical spine to demonstrate the final position of the implanted leads within the posterior epidural space (SUNA patient, 42 months follow-up)

All patients underwent a 2-weeks trial of HF10 SCS. One or two octad leads were used according to the surgeon’ preference. If the trial was deemed “successful”, i.e. at least 50 % decrease in headache intensity and/or frequency, a permanent implant was then performed under general anaesthesia. The trial implant technique entails epidural lead placement through a small skin incision under local anaesthesia supplemented by conscious sedation. Under fluoroscopic control, a 14-gauge Tuohy type needle was introduced at the upper thoracic level and advanced into the epidural space. One or two-eight-contact cylindrical leads were advanced cranially in the posterior epidural space, until the distal tip was approximately at the C_2_ vertebral level (Fig. 1). The leads were anchored and sutured to the supraspinal ligament/paravertebral muscle fascia, connected to temporary extensions, tunneled 30 cm under the patient’s skin and connected to a temporary external stimulator for the duration of the trial period. Multiple stimulation programmes (frequency: 10 kHz; amplitude: 1.5–4 mA; pulse width: 30 μs) were provided to target the dorsal columns in the area corresponding to C_2_-C_3_ vertebral level.

Subjects with a successful trial were subsequently implanted with an internal pulse generator. The proximal end of the tunnelled lead(s) were connected to new sterile extensions which provided connection to the implantable pulse generator (IPG) (Senza™ system, Nevro Corp., Menlo Park, USA), which was implanted subcutaneously either in the anterior abdominal wall or the gluteal region.

### Data collection and follow-up

Baseline headache characteristics, including headache frequency, severity, duration and medications consumption, were collected retrospectively from patients’ records, headache charts, outpatient visits and telephone clinics. Baseline headache-related disability scores were gathered from HIT-6 questionnaires. After the operation, patients were asked to fill a headache diary. More recently CM- and a TACs-specific diaries were designed in our headache centre, hence a daily headache diary and an attack diary were respectively given to CM and TACs patients. HIT-6 questionnaires were administered to patients before scheduled follow-ups. The outcomes used to assess the effectiveness of HF10 SCS for the migraine patients include: change in number of headache days and migraine days, defined according to International guidelines [20] together with change in headache severity and reduction in abortive treatment consumption. For the TACs patients we assessed change in number of daily attacks, change in duration and severity of the attacks and change in medication use. Adverse events were collected from patients’ notes.

## Findings

### Migraine

Four patients (3 males and 1 female; age at the time of implant: 42–55 years old) with refractory CM had a successful stimulation trial and underwent a full implant of HF10 SCS. Table 1 summarises their baseline pre-operative and post-operatively clinical outcomes. All were overusing medication at the time of the surgery. As part of our routine headache management, patients 1, 2 and 3 withdrew analgesics for more than 2 months without noticeable improvements, whereas patient 4 declined to discontinue the use of injectable sumatriptan. Three out of four patients had failed to respond to at least two sets of Botulinum toxin type A (Botox®) according to the PREEMPT paradigm [18]. Patient 1, after having failed numerous medical treatments, was treated with occipital nerve stimulation (ONS) in 2008. The therapy was initially effective in reducing the severe migraine attacks but not the background headache. Unfortunately after 3 years the benefits of ONS declined, despite multiple reprogramming attempts, therefore the device was explanted in September 2012. Patients 1, 2 and 4 have not been taking any oral preventive medicines since the implant of HF10 SCS. Patient 3 was on a stable dose of pregabalin 300 mg/day and nortriptyline 30 mg/day at baseline, with marginal benefit.

**Table 1:** Clinical characteristics of chronic migraine patients pre- and post-high frequency cervical spinal cord stimulation treatment

	Duration of chronic headache at time of implant	Number of preventive treatments failed	Follow-up after surgery (months)	Number of headache days/month		Number of migraine days/month		Average headache severity		Analgesia consumption (days/month)		HIT-6 (score)	
				Pre	Post	Pre	Post	Pre	Post	Pre	Post	Pre	Post
1	14 years	7 (including ONS)	40	Daily	5–7	8–12	2–3	7/10	7/10	8–12 (PCM, COD)	2–3 (PCM, COD)	76	65
2	3 years	9 (including Botox®)	25	Daily	10	16–24	7	7/10	4/10	15 (COD, IBP, TRA)	4 (TRA, ALM)	68	51
3	3 years	8 (including Botox®)	24	Daily	6–7	16	6–7	6/10	6/10	Daily (PCM, COD)	6–7 (PCM, COD)	74	64
4	10 years	6 (Not willing to have Botox®)	12	Daily	6	8–12	3	8/10	4/10	Daily (SUM injections)	1 (SUM injection)	73	44

*ALM* almotriptan, *COD* codeine, *HIT-6* headache impact test, *IBP* ibuprofen, *ONS* occipital nerve stimulation, *PCM* paracetamol, *SUM* sumatriptan, *TRA* tramadol

At an average follow-up of 25.3 months (range: 12–40 months) post-implant, all patients reported remarkable benefits, with at least a 50 % reduction in monthly number of headache days and migraine days, which led them to revert to an episodic headache pattern (<15 headache days/month). This was associated with a marked reduction in number of days where patients needed to take abortive medications. Patient 3 was also able to discontinue his preventive treatments few months after device activation. At baseline, all patients were classified as severely disabled according to their HIT-6 scores. At the latest available post-operative follow-up, the HIT-6 scores were reduced in all patients; the reduction was meaningful in two patients that rated their headache disability as of “little and moderate impact” (patient 2 and 4). All patients rated their overall percentage of headache improvement between 50–100 %. Most patients reported headache improvement a few days after the HF10 SCS trial was commenced (patients 1, 3 and 4), whereas in patient 2 different stimulation programmes were tried before achieving the maximum benefit.

Continuous stimulation was used in all cases. Unintentionally, patients 1 and 2 switched off the device on few occasions and experienced an exacerbation of their headache that settled within approximately 1 to 2 weeks after the device was switched on again. Stimulation programmes (10 kHz; 30 μs; 1.5–4 mA) were provided to target the dorsal columns in the area corresponding to C_2_–C_3_ x-ray vertebral body. Patients 1 and 3 had one lead implanted and benefited from activation of the top two contacts. Patients 2 and 4 had two leads implanted: one at the midline and one more laterally towards the side mostly affected by the headache: they both benefited from alternating between the two stimulation programmes, one using the top two contacts of one lead and the other using the top two contacts in both leads. None of the patients reported any stimulation-induced sensation while the device was on. There were no reports of serious device-related adverse events. Patient 1 in June 2013 experienced a worsening of his headache: high impedances on all contacts suggested system malfunction, which was caused by lead breakage. A surgical lead replacement led to an improvement of the headache within 1 week of device reactivation. Patient 3 and 4 initially reported mild to moderate discomfort at the IPG pocket, which settled respectively after 3 and 4 months.

### SUNA syndrome

Two patients with chronic SUNA were treated with HF10 SCS. The first patient was a 50 year-old woman with an 8-year history of chronic left-sided SUNA syndrome according to the IHS criteria (patient 1, Table 2). The headache was interfering significantly with her quality of life and had forced her to quit her job. In April 2013 she had a successful trial of HF10 SCS, which led to permanent IPG (10 kHz; 30 μs; 0.4–1 mA). The therapy provided a complete resolution of the SUNA attacks for 8 months, a part from seldom episodes of cranial autonomic symptoms without headache. Subsequently the SUNA attacks relapsed and at 28 months of follow-up they have been occurring 10–20 times per day, lasting 2–30 s on a visual rating scale (VRS) between 5–7/10. There have been no device-related adverse events. The patient rated the overall improvement of the HF10 SCS at about 70 %. She has not been taking any preventive medication since the implant. She has been able to return to work initially part-time and then full time at the latest available follow-up visit.

**Table 2:** Clinical characteristics, medical treatments and MRI outcome in Trigeminal autonomic cephalalgias patients treated with high frequency cervical spinal cord stimulation treatment

	Patient 1	Patient 2	Patient 3
Diagnosis	Chronic SUNA	Chronic SUNA	CCH
Side of pain	Left	Right	Left
Site of pain	V1-V2-V3-C2	V3-V2-V1	V1
Pain character	Stabbing	Stabbing	Stabbing
Attack duration	2–600 s	180–300 s	40–180 min
Attack frequency (daily)	30–50	50–60	1–10
Pain severity (VRS)	8–10/10	10/10	10/10
Ipsilateral autonomic features	- Ptosis - Rhinorrhoea - Facial oedema - Facial redness	- Blocked nose - Rhinorrhoea - Facial oedema - Facial redness	- CI - Lacrimation - Rhinorrhoea - Facial sweating
Migrainous symptoms	Yes	Yes	No
Cutaneous triggers	Yes	No	No
Background pain	Yes	Yes	Yes
Effect of indometacin	None	None	None
Failed treatments	- Lamotrigine - Carbamazepine - Oxcarbazepine - Prednisolone - Topiramate - Gabapentin - Pregabalin - Duloxetine - Amitriptyline - Flunarizine	- Lamotrigine - Carbamazepine - Gabapentin - Pregabalin - Amitriptyline^a^	- Oxygen - Sumatriptan sc - Verapamil - Lithium - Prednisolone - Gabapentin - Pregabalin - Topiramate - Melatonin - Indometacin - Baclofen - Sodium Valproate - Levetiracetam - GONB - MCNB - SPG block - IV lidocaine
MRI brain	Normal	Normal	Normal

*CCH* chronic cluster headache; *CI* conjunctival injection, *GONB* greater occipital nerve block, *IV* intravenous, *MCNB* multiple cranial nerve block, *SC* subcutaneous, *SPG* sphenopalatine ganglion, *SUNA* short-lasting unilateral neuralgiform headache attacks with autonomic symptoms, *VRS* verbal rating scale, *V1* ophthalmic trigeminal division, *V2* maxillary trigeminal division, *V3* mandibular trigeminal division

^a^Patient 2 declined any further pharmacological treatments

The second patient was a 43-year old woman with a 12-year history of a headache condition that fulfils the IHS criteria for chronic migraine. Seven years ago a new headache began. The condition was initially occurring with an episodic pattern though after 6 months from the onset, it became chronic, without remission periods. The headache fulfilled the IHS criteria of chronic right-sided SUNA syndrome (patient 2, Table 2). In July 2012 she underwent a successful trial and subsequently a full implant of HF10 SCS (10 kHz; 30 μs; 0.4–2 mA). During the first 16 months of treatment, she reported only two SUNA attacks/month lasting 3–5 min, moderate in intensity (VRS 6/10). The attacks subsided once the stimulator was reprogrammed again. Between month 16 and month 37, two reprogramming sessions were required to maintain complete headache relief. After 42 months, she continues to report almost complete resolution of both the SUNA attacks and the chronic migraine, with no adverse events.

### Cluster headache

One patient with CH was treated with HF10 SCS. He was a 41-year-old man with a 7-year history of left-sided CCH according to the IHS criteria, which became refractory to medical treatments (patient 3, Table 2). He was offered a trial with occipital nerve stimulation (ONS) in March 2011. He became headache-free for 6 months following the implant before the CH relapsed. Despite re-programming, the ONS failed to improve his condition and it was discontinued. In view of the severity of his condition, he was trialled with HF10 SCS (10 kHz; 30 μs; 0.5–1 mA). This was successful, leading to full implant. He was then headache free for 9 months. However the CH attacks gradually came back, occurring at the same frequency and severity of the baseline, but with a significant reduction in duration: from 40–180 min at baseline, to 20–35 min with active HF10 SCS. He rated the improvement at 50 %. Since the implant, trials of sodium valproate and levetiracetam were implemented to improve the condition further. Unfortunately those were not tolerate even at low doses and therefore discontinued. The device was switched off in one occasion for 1 month, leading to a worsening of the CH. A lead revision was necessary after a lead migration occurred 11 months after the surgery.

## Discussion

The management of patients with primary headache conditions refractory to pharmacological treatments remains challenging. ONS is currently considered the surgical treatment of choice for refractory CM and chronic TACs [21]. However its acceptance has been limited by the outcome of methodologically weak RCTs. It is virtually impossible to design good, blinded controlled studies for ONS because the therapy produces paresthesia. A concern with ONS therapy is complication rates. Long-term follow-up of the largest ONS clinical trial conducted in CM showed a total of 183 device-/procedure related adverse events during the study, with 29 lead migrations. Other hardware-related complications include IPG migration, lead breakages or fractures, lead or extension disconnection or malfunction, programmer malfunction or IPG malfunction. Eighty-five adverse events (40.7 %) required surgical intervention and 18 adverse events (8.6 %) required hospitalization [22]. The high rate of hardware-related complications and surgical revisions has recently led the European Notified Body to remove intractable CM as an indication for ONS therapy [23].

HF10 SCS is a paraesthesia-free system, which has been shown to inhibit evoked afferent nociceptive inputs by modulating wide-dynamic range neuronal activity in the spinal cord of different animal models [24]. HF10 SCS has shown remarkable clinical efficacy in human refractory back pain [13, 15]. When applied to the cervical epidural space, it has been also reported as safe and effective in a series of subjects with upper limb neuropathic pain [14]. We postulate that stimulation directly to the dorsal columns at the level of C2-C3 vertebral bodies with HF10 SCS may modulate the trigeminocervical complex and in turn have a therapeutic effect on primary headaches.

This case series provides initial evidence of effectiveness of HF10 SCS in the management of refractory primary headache disorders. In the treated CM group we observed a significant reduction in headache and migraine days, abortive treatment intake and headache-related disability. The four CM patients all reverted from chronic headache pattern to episodic. Arcioni *et al* reported promising results using HF10 SCS in a small group of CM patients with medication overuse. Fifty percent of patients reported at least a 30 % reduction in headache days at 6 months. Furthermore HF10 SCS therapy led to reduction of analgesia consumption and disability headache-related in responders [16]. Taken together, these data indicate cervical HF10 SCS as a new, promising treatment in refractory CM. A caveat of the outcome analysis for both studies could be the contribution of the medication withdrawal effect. However in our study, three out of four patients had undergone pre-implantation medications withdrawal without any noticeable change in the headache pattern, suggesting that the improvement is more likely related to HF10 SCS, rather than to the reduction in abortive medications intake.

To date there are no published data on effectiveness of HF10 SCS in TACs. However, high cervical low frequency SCS has shown promising outcome in a small series of seven refractory CCH patients [25]. At a median follow-up of 12 months, the Authors observed a meaningful improvement of the headache in all the implanted patients, allowing reduction in medications intake. Three chronic refractory TACs patients were treated with HF10 SCS in this case series. We observed long-lasting and meaningful benefits in two SUNA patients. The CCH patient, after an initial pain-free period of a few months, experienced reduction in the duration but not in the intensity and frequency of the attacks as the final outcome. The CCH case had been refractory to many other treatments including ONS, thus the partial improvement achieved was been rated as meaningful by the patient.

HF10 SCS may have potential clinical and technical advantages compared to ONS. The HF10 SCS post-implantation therapeutic effect seems to occur almost immediately. A rapid response was noted also in the HF10 Arcioni *et al* study [16] and in the Wolter *et al* CCH series, using a low frequency stimulation device [25]. In contrast, the ONS effect seems to take several weeks or months to develop [9, 26]. This suggests that a short trial treatment may be predictive of outcome with central neuromodulation techniques, but not with peripheral ONS. However, a very high positive trial rate, like that seen in our series (100 %) as well as in the Arcioni *et al* study [16], could obviate the need for a stimulation trial, given the additional risks and inconvenience to patients and costs to the health care system [7]. The faster post-implant response of cervical high- and low-frequency SCS compared to ONS, may reflect the anatomical difference in treatment target, suggesting that direct stimulation of the spinal cord may recruit the trigemino-cervical complex more efficiently.

In this case-series two out of the seven patients (29 %) developed hardware-related complications that required additional surgery. Although based on very small numbers, this complication rate is in line with the one reported by Arcioni *et al* (24 %) [16], and is lower than reported in ONS studies (40.7 %) [22]. The epidural, axial placement of HF10-SCS leads could be expected to have a lower complication rate than see with ONS leads which are placed at the mobile cranio- cervical junction.

Patients tolerated the paraesthesia-free stimulation well. Historically, the presence of stimulation-produced paresthesia has been considered essential for achieving analgesia in headache neurostimulation studies [11]. The effectiveness of a paresthesia-free neurostimulation system implies that paresthesia sensation may not be required to achieve analgesia [27]. Since the paraesthesia sensation associated with traditional low-frequency ONS is at times considered uncomfortable and may lead to therapy interruption, using a paresthesia-free device may be better accepted by patients.

Limitations of this case series include its retrospective nature and the absence of a control group, raising the possibility that the effect of HF10 SCS in our patients might be attributable to placebo or natural history. Several observations suggest differently: a protracted chronic phase, lack of response to several other treatments, relatively high response rate, sustained long-term improvement, and the rapid deterioration of the headache after the device was switched off or had technical failures.

In this small case series of medically refractory primary chronic headache subjects, the long-term HF10 SCS therapy was well tolerated and beneficial, with an acceptable safety profile (at least comparable with existing implantable neurostimulation devices). In view of the considerable need for improved treatments for this highly disabled group of patients, further prospective research in this field is warranted.

## Abbreviations

CCH, chronic daily headache; CDH, chronic daily headache; CM, chronic migraine; HC, hemicrania continua; HF10 SCS, 10 kHz spinal cord stimulation; HIT-6, Headache Impact Test; IHS, International Headache Society; IPG, implantable pulse generator; ONS, occipital nerve stimulation; PH, paroxysmal hemicrania; RCTs, randomized controlled trials; SUNA, Short-lasting Unilateral Neuralgiform headache attacks with Autonomic symptoms; SUNCT, Short-lasting Unilateral Neuralgiform headache attacks with conjunctival injection and tearing; TACs, trigeminal autonomic cephalalgias; VRS, visual rating scale
